# Clustering of disulfide-rich peptides provides scaffolds for hit discovery by phage display: application to interleukin-23

**DOI:** 10.1186/s12859-016-1350-9

**Published:** 2016-11-23

**Authors:** David T. Barkan, Xiao-li Cheng, Herodion Celino, Tran T. Tran, Ashok Bhandari, Charles S. Craik, Andrej Sali, Mark L. Smythe

**Affiliations:** 1 Protagonist Therapeutics, Inc., 521 Cottonwood Drive, Suite 100, Milpitas, CA 95035-74521 USA; 2 Institute for Molecular Bioscience, The University of Queensland, St. Lucia, Qld 4072 Australia; 3 Department of Bioengineering and Therapeutic Sciences, University of California, San Francisco, San Francisco, CA 94158 USA; 4 Department of Pharmaceutical Chemistry, University of California, San Francisco, San Francisco, CA 94158 USA; 5 California Institute for Quantitative Biosciences (QB3), University of California, San Francisco, San Francisco, CA 94158 USA

**Keywords:** Disulfide-rich peptides, Drug discovery, Clustering, Phage display, Interleukin-23, Knottins, Structure conservation

## Abstract

**Background:**

Disulfide-rich peptides (DRPs) are found throughout nature. They are suitable scaffolds for drug development due to their small cores, whose disulfide bonds impart extraordinary chemical and biological stability. A challenge in developing a DRP therapeutic is to engineer binding to a specific target. This challenge can be overcome by (i) sampling the large sequence space of a given scaffold through a phage display library and by (ii) panning multiple libraries encoding structurally distinct scaffolds. Here, we implement a protocol for defining these diverse scaffolds, based on clustering structurally defined DRPs according to their conformational similarity.

**Results:**

We developed and applied a hierarchical clustering protocol based on DRP structural similarity, followed by two post-processing steps, to classify 806 unique DRP structures into 81 clusters. The 20 most populated clusters comprised 85% of all DRPs. Representative scaffolds were selected from each of these clusters; the representatives were structurally distinct from one another, but similar to other DRPs in their respective clusters. To demonstrate the utility of the clusters, phage libraries were constructed for three of the representative scaffolds and panned against interleukin-23. One library produced a peptide that bound to this target with an IC_50_ of 3.3 μM.

**Conclusions:**

Most DRP clusters contained members that were diverse in sequence, host organism, and interacting proteins, indicating that cluster members were functionally diverse despite having similar structure. Only 20 peptide scaffolds accounted for most of the natural DRP structural diversity, providing suitable starting points for seeding phage display experiments. Through selection of the scaffold surface to vary in phage display, libraries can be designed that present sequence diversity in architecturally distinct, biologically relevant combinations of secondary structures. We supported this hypothesis with a proof-of-concept experiment in which three phage libraries were constructed and panned against the IL-23 target, resulting in a single-digit μM hit and suggesting that a collection of libraries based on the full set of 20 scaffolds increases the potential to identify efficiently peptide binders to a protein target in a drug discovery program.

**Electronic supplementary material:**

The online version of this article (doi:10.1186/s12859-016-1350-9) contains supplementary material, which is available to authorized users.

## Background

In the past decade of drug discovery, peptide-based drugs have gathered momentum as a class of therapeutics, with their global market impact expected to increase significantly in the future [1]. Previously, the spectrum of available drugs consisted primarily of small molecules that target deep binding pockets on proteins to inhibit enzyme function. However, small molecules are generally not well-suited for binding to large, flat surfaces on a protein to inhibit protein-protein binding, a process that is critical for treating many human diseases [2]. In addition, small molecules frequently lack binding specificity, a disadvantage that can lead to failure in the development pipeline or to adverse side effects, even among drugs on the market [3]. In contrast, biologic-based drugs, such as monoclonal antibodies, have been found to be highly specific and effective blockers of protein-protein interactions, and their clinical use has transformed medicine over the past decade. Despite the growing success of antibody-based drugs, they do have several limitations. They are large and complex macromolecules that need to be delivered by injection, have long circulating half-lives with little ability to control drug levels in patients precisely, leading to safety consequences, and lack durability with patients losing response due to immunogenicity.

In contrast to proteins, peptides, as defined in this study, consist of up to 50 amino acid residues and lack a hydrophobic core [4]. The simplest peptides are linear and disordered, assume structure only upon binding to a protein, and are prone to degradation by host proteases. Thus, peptide drug design strategies often seek to engineer structure into the peptide [5]. These approaches include induction of secondary structure such as β-turns, α-helices and β-hairpins [6]; head-to-tail cyclization [7, 8]; and incorporation of non-standard amino acid residues, as in peptoids [9]. Of particular interest is creating a peptide fold through formation of disulfide bonds between cysteine residues that are distant from each other in the peptide sequence. In this study, we define a disulfide-rich peptide, or DRP, as a peptide consisting of up to 50 residues and incorporating between one and four disulfide bonds. DRPs often do possess a hydrophobic core, although this region is generally occupied by the bonded cysteines.

Many of the desirable properties of therapeutic compounds found in DRPs are demonstrated by their biological functions. They frequently adopt the ‘knottin’ motif in which six or more cysteines form disulfide bonds in an interlocking arrangement, often incorporating head-to-tail cyclization [10]. The knottin motif consists of several forms, each with its own possible secondary structure elements; these forms include the inhibitory cysteine knot (ICK), in both its classic and cyclotide arrangements, as well as the cysteine-stabilized αβ (CSαβ) fold [11, 12]. Knottins have diverse functions ranging from plant defense [13] to incapacitating prey when expressed as toxins in venomous animals [14], and have been reported to show low-immunogenic potential [15], which avoids challenges often presented by other biologics, such as antibodies. Another fold class is small β-hairpins stabilized both by the standard backbone hydrogen-bond patterns as well as one or more disulfide bonds linking the paired β-strands. These hairpins are often natural protease inhibitors [16], or can be converted to such with simple modifications [17]. Other examples of DRPs in nature include anti-microbial defensins [18], small conotoxins [19], and insulin [20].

Disulfide bonds stabilize the fold of a peptide by decreasing the entropy of the system proportionally to the number of residues between the linked cysteines [21, 22]. This increased stability confers beneficial properties necessary in a drug, including enhanced potency, selectivity, permeability, thermal stability, resistance to denaturation at low pH, protection against proteolytic attack [23], and in some instances increased activity when delivered orally [24–27]. Disulfide bonds may lock the molecule into a conformation that is complementary to a protein target [28], providing an opportunity to engineer the surface with new functionality while maintaining the fold. For example, a number of studies have grafted the binding surface of a protein onto a DRP scaffold, resulting in a molecule that retains the advantages of DRPs while reproducing the binding properties of the original protein [29, 30]. Current drugs on the market incorporating disulfide bonds include insulin, orally delivered linaclotide for treating inflammatory bowel syndrome [31], ziconotide for treatment of pain [32], and pramlintide as an adjunct therapy for type II diabetes [33].

While DRPs are used as starting points for designing inhibitors of protein-protein interactions, modifying the DRP sequence to enable specific binding to a desired protein target remains a challenge. One potential solution is phage display, which can sample up to 10^12^ unique protein sequences and allows for selection of those that bind the target [34]. In one form of this experiment, a DNA library encoding a peptide, with some or all of the codons randomized, is ligated into a phage plasmid in a gene encoding for a coat protein, resulting in a library of phage expressing diversified peptide sequences on their surface. The library is then introduced to an immobilized protein target and screened in a procedure referred to as ‘panning’. Phage particles with peptides that bind the immobilized target are selected over those that do not, which are subsequently washed away. The enriched population of clones expressing binding peptides is then amplified and the process is repeated in an iterative panning and amplification process. Finally, the selected phage clones, referred to as hits, are sequenced and the peptides corresponding to those sequences are synthesized and assayed to confirm binding or in some cases functional activity. A number of studies have used DRPs as phage library scaffolds [35]; we have recently reported the rational design and development of potent IL-6 compounds using this method [36].

A drawback in phage display is that a single phage library may yield no hits when panned against a target, regardless of the sequences displayed in the library, due to (i) the possibility that none of the generated sequences is complementary to the target or (ii) the inability to select rare and weakly active phage clones in a large pool of inactives. Therefore, we hypothesize that the probability of obtaining a hit increases if multiple phage libraries encoding structurally distinct scaffolds are used. As more unique scaffolds are panned, it is increasingly likely that at least one of them will result in a sequence with sufficient affinity for binding the target. The challenge then becomes the selection of DRPs to use as phage library scaffolds. To reduce the odds of creating redundant phage libraries, we propose that the scaffold DRPs should be as structurally distinct, and should cover as large a fraction of known DRP folds, as possible.

One solution to this challenge is to group DRPs according to structural similarity and select a representative from each DRP cluster, thus guaranteeing that the representative DRPs are structurally distinct. The set of representatives should be small enough to make it experimentally tractable to construct a phage library using each representative as a scaffold. Each cluster should include as many DRPs as possible, thus allowing for a maximum estimation of the fraction of total DRP structural diversity covered by the representative peptides. Finally, the method should be automated so that the clustering can be updated as more DRP structures are solved and added to the Protein Data Bank (PDB). However, the number of structural folds into which DRPs can be clustered is not known, so there is no guarantee that all of these properties can be achieved. There have been previous attempts to perform such clustering, but they were either focused on a subset of DRP fold classes or required significant manual intervention [37–39].

Here, we describe an automated DRP clustering protocol that incorporates structural similarity and disulfide-bond conservation to group related DRPs, accompanied by a metric to select a representative member from each cluster for use as a scaffold for phage display. The method was applied to all known DRP structures in the PDB. The 20 most populous clusters contained 85% of all structurally known DRPs. By examining the clusters, we analyzed the degree to which DRPs can be grouped together, examined how sequence conservation varies within each cluster, and assessed whether our approach has produced a set of scaffolds structurally distinct from each other but similar to other DRPs in their clusters. Moreover, to demonstrate the utility of the method, we designed libraries based on three of the 20 scaffolds and panned them against the human interleukin-23 cytokine protein, resulting in a low micromolar hit from one of the three libraries.

Previous approaches have been successful in engineering into a DRP the ability to bind a target, either through phage display [35], grafting the exact binding surface of a protein known to bind the target [29], or a combination of the two [40]. Based on these successes, we propose that using phage display to pan multiple scaffolds possessing maximally structurally diverse binding surfaces greatly increases the likelihood of finding an initial hit against a target. Separately, we also hypothesize that, while DRP folds found in the PDB are likely not completely representative of all DRP folds found in nature, they do represent a large fraction, possibly even the majority of such folds, and thus our scaffolds are representative of a similarly large fraction of possible DRP structural diversity. Therefore, especially considering their favorable chemical and biological stabilities, the phage libraries for these 20 representatives will be a valuable resource for discovering DRPs interacting with protein targets.

## Results

### Grouping DRPs into fewer clusters as protocol proceeds

#### Pipeline overview

**Fig. 1:**
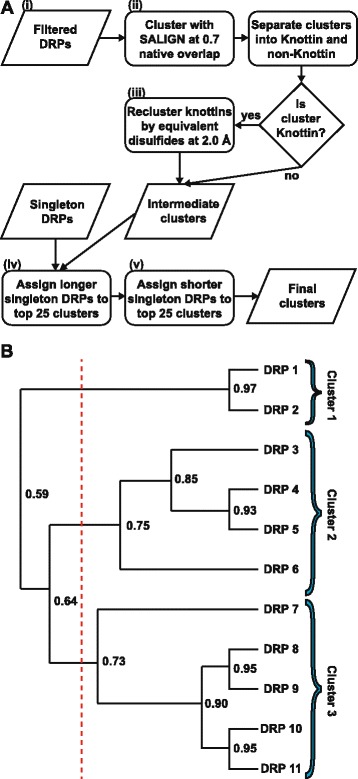
Protocol details**. a** Pipeline workflow. **b** Example of hierarchical clustering using toy data, portrayed as a tree where the leaves are DRPs and each inner node represents a cluster containing all DRPs in the sub-tree rooted at that node. Numbers at the branch point are the values of the distance metric when calculated across the two sub-trees that are being merged at the inner node. The red line is the empirically selected cutoff (here, 0.7); all sub-trees to the right of this cutoff represent the final clusters

Our computational pipeline consisted of five steps: (i) filtering, (ii) hierarchical clustering using native overlap as the distance metric, (iii) reclustering knottins using disulfide distance as the distance metric, (iv) re-assigning longer singletons, and (v) re-assigning shorter singletons (Fig. 1a; Methods).

### Filtering identical DRPs

The PDB was searched for individual chains with fewer than 50 residues and between one and four annotated disulfide bonds, resulting in 1,411 DRPs fitting these criteria. This initial dataset was filtered further to remove identical DRPs, including 292 identical insulin chains, resulting in 806 representative structures (Fig. 1a, step i).

### Native overlap clustering and cutoff determination

**Fig. 2:**
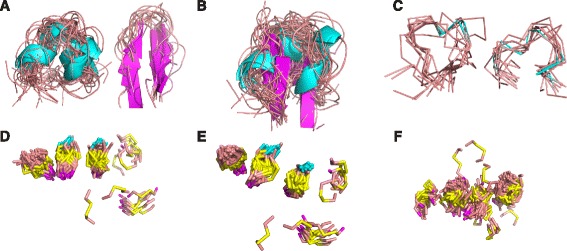
Determination of clustering cutoffs. *Top row*: example of resulting clusters following the initial native overlap hierarchical clustering step. Each image represents a different cutoff applied for determining the final clusters for that step. These images informed the decision of which cutoff to impose in the final protocol. **a** Conotoxin and small hairpin clusters at the native overlap cutoff of 0.7, which was ultimately selected as the final cutoff. **b** At a cutoff of 0.6, the same conotoxin and small hairpin DRPs were assigned to the same cluster despite assuming different secondary structures. **c** At a cutoff of 0.8, conotoxin DRPs were assigned to separate clusters despite each cluster fold consisting of circular loops and short helical regions. Bottom row: example of a resulting cluster following the knottin reclustering step, with each image representing the knottin cluster containing the most DRPs after applying a different cutoff. Only the disulfide bonds in the DRPs are displayed, in yellow. The cutoffs assessed were (**d**) 2.0 Å RMSD, (**e**) 1.5 Å RMSD, and (**f**) 2.5 Å RMSD. 2.0 Å was selected as the optimal cutoff and used in the final protocol

The 806 representative DRPs were clustered using native overlap as the distance metric in an average-linkage hierarchical clustering algorithm (Fig. 1b; Methods). The algorithm terminated when the smallest average native overlap between any two clusters was below a cutoff. The value of this cutoff was determined by trial-and-error, selecting the optimal cutoff of 0.7 through visualization of clusters with 3D structure viewing software (Fig. 2a). A cutoff of 0.6 was considered, but it resulted in assigning DRPs with clearly different folds to the same cluster; for example, small β-hairpins, which have tight turns in between successive β-strands, clustered with conotoxins, which are similar to hairpins in size but have rounded turns connecting loops or helices (Fig. 2b). On the other hand, a cutoff of 0.8 was too stringent, assigning DRPs with very similar structures into different clusters (Fig. 2c). The average-linkage hierarchical clustering step using the selected native overlap cutoff of 0.7 grouped the 806 DRPs into 178 clusters (Fig. 1a, step ii).

### Knottin reclustering and cutoff determination

Peptides were annotated with SCOP identifiers [41]. DRPs with the same SCOP fold identifiers were generally in the same clusters, validating the clustering procedure (Additional file 1: Table S1). However, the 260 ‘knottins’ (SCOP ID g.3) were classified into 15 distinct clusters, due to their varied loop lengths. In phage display experiments, loop lengths may be varied as part of the library design, and the core structures of the scaffolds are of greater importance. Therefore, knottins were reclustered by their core disulfide bond structure only (Fig. 1a, step iii), as follows.

**Fig. 3:**
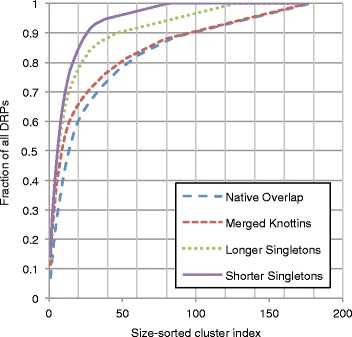
Cluster DRP coverage. Clusters were sorted by size from most to least populated and each cluster was assigned an index starting with 1. At each index *i*, the cumulative number of DRPs in that cluster and all clusters with index less than *i* was calculated and divided by the total number of DRPs in the dataset, resulting in the coverage. Coverage as a function of index is displayed. Coverage curves are shown after completion of successive steps of the procedure (*red*: initial clustering; *green*: knottin reclustering; *purple*: longer singleton post-processing; *blue*: shorter singleton post-processing)

Clusters containing four or more DRPs annotated with the knottin fold were given as input to the average-linkage hierarchical clustering algorithm, here using the distance between equivalent disulfide bonds as the distance metric (Methods). A disulfide distance cutoff of 2.0 Å was again selected by trial-and-error. This cutoff resulted in high structural overlap of disulfide bonds across DRPs in the knottin clusters (Fig. 2d) with a separation of ~1.8 Å between consecutive groups of bonds in the most populated cluster despite 91 members being present. The cutoff of 1.5 Å resulted in a similar separation, but here, only 64 members were in the most populated cluster (Fig. 2e), resulting in suboptimal lower coverage. The cutoff of 2.5 Å led to 131 members in the most populated cluster, but there was no clear visual separation apparent in consecutive groups of disulfide bonds (Fig. 2f). This cutoff would likely render selection of a representative scaffold problematic, as there would be no DRP in the cluster that possessed a set of disulfide bonds structurally equivalent to all other members of the cluster. The optimal cutoff of 2.0 Å reduced the number of clusters containing four or more knottins from 15 to 4 (Additional file 1: Table S2). Together with all non-knottin clusters produced in step ii, there were 176 intermediate DRP clusters (Fig. 3).

### Singleton reassignment

Next, we observed that some DRPs in less-populated clusters had native overlaps above 0.7 when aligned to peptides in clusters with more members. However, the hierarchical nature of the procedure grouped the most similar DRPs together with each iteration; this process sometimes resulted in a DRP being grouped with its closest neighbor in a small cluster even if there was another more populated cluster containing members similar to that DRP. Provided the DRP aligned to at least one peptide in the larger cluster at a native overlap of 0.7, the DRP was reassigned (i.e.*,* a singleton) to the larger cluster. This post-processing refinement increased the sizes of the most-populated clusters (Fig. 1a, steps iv-v), and reduced the total number of clusters from 176 to 81 (Fig. 3). The full composition of all clusters is available in Additional file 1: Table S3.

### DRP majority representation in 20 structure folds

**Fig. 4:**
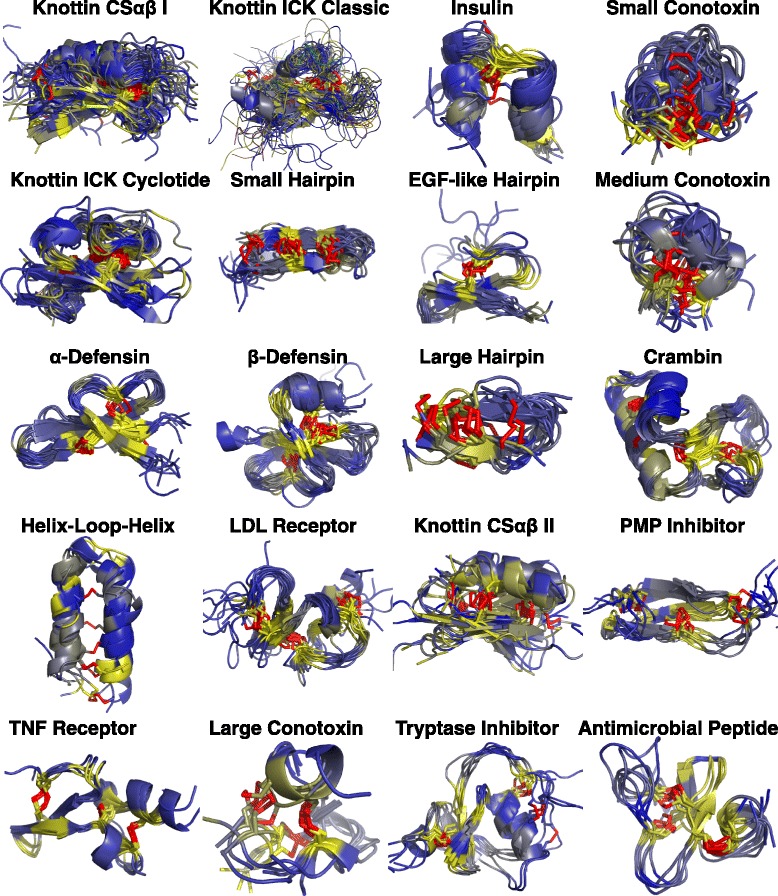
Cluster visualization. The top 20 clusters by size are displayed. Singleton DRPs are removed for clarity. DRPs are colored according to sequence conservation within the cluster, ranging from *yellow* (high conservation) to *gray* (moderate) to *blue* (low conservation). Disulfide bonds are shown in *red*

A primary goal of the clustering procedure was to identify a small number of representative DRPs, as this goal balanced a number of peptide scaffolds large enough to cover a significant fraction of DRP structure space but small enough to be experimentally tractable in phage display experiments. The method resulted in 84.5% of DRPs in the PDB being assigned to the top 20 most populated clusters (Fig. 3). Although 81 distinct DRP folds were identified, the least populated 61 clusters each contained only nine or fewer DRPs, with 43 of these clusters containing a single peptide. It is feasible to construct 20 phage libraries, which would be structurally representative of nearly 85% of all DRPs whose structures have been solved. Images of these top 20 clusters ranked by membership are presented in Fig. 4.

### Structural diversity of cluster representatives

From each of the top 20 clusters, the DRP with the largest average native overlap to all other members of that cluster was identified; this DRP was selected as the representative member of the cluster and considered a potential scaffold for phage display. One goal of the study was to select representative DRPs that were structurally distinct from the other representatives, which was assessed by the average native overlap between the representatives. All representative DRPs had an average native overlap to other DRPs in their clusters of greater than 0.64; the median value across the 20 clusters was 0.77 (Table 1, diagonal values). In contrast, the median native overlap for pairs of representatives was only 0.39 (Table 1, off-diagonal values), indicating that the clustering procedure indeed resulted in a structurally diverse set of DRP scaffolds that were truly representative of the majority of known DRP structure space. Additional details of each cluster are presented in Table 2.

**Table 1:** Matrix of structural diversity across clusters

	**1**	**2**	**3**	**4**	**5**	**6**	**7**	**8**	**9**	**10**	**11**	**12**	**13**	**14**	**15**	**16**	**17**	**18**	**19**	**20**
**1**	**.73**	.64	.42	.25	.67	.39	.50	.31	.67	.42	.31	.41	.53	.34	.69	.39	.39	.33	.39	.58
**2**	.64	**.76**	.39	.27	.73	.42	.58	.39	.48	.47	.33	.24	.42	.47	.70	.70	.50	.27	.45	.70
**3**	.42	.39	**.85**	.52	.34	.38	.48	.67	.40	.36	.38	.41	.50	.32	.46	.33	.39	.57	.32	.33
**4**	.25	.27	.52	**.77**	.31	.53	.47	.75	.33	.31	.67	.26	.31	.26	.32	.24	.31	.63	.25	.33
**5**	.67	.73	.34	.31	**.79**	.38	.55	.38	.37	.42	.38	.37	.34	.45	.69	.61	.44	.45	.23	.77
**6**	.39	.42	.38	.53	.38	**.71**	.74	.53	.50	.33	.65	.22	.28	.34	.54	.45	.36	.47	.34	.37
**7**	.50	.58	.48	.47	.55	.74	**.66**	.53	.33	.39	.58	.26	.34	.45	.61	.58	.50	.47	.27	.57
**8**	.31	.39	.67	.75	.38	.53	.53	**.80**	.40	.31	.50	.30	.41	.37	.39	.27	.42	.63	.25	.37
**9**	.67	.48	.40	.33	.37	.50	.33	.40	**.91**	.72	.30	.33	.38	.39	.50	.55	.39	.37	.43	.63
**10**	.42	.47	.36	.31	.42	.33	.39	.31	.72	**.91**	.25	.50	.36	.42	.56	.42	.47	.33	.34	.28
**11**	.31	.33	.38	.67	.38	.65	.58	.50	.30	.25	**.80**	.20	.19	.29	.39	.33	.31	.47	.25	.33
**12**	.41	.24	.41	.26	.37	.22	.26	.30	.33	.50	.20	**.98**	.41	.35	.30	.30	.28	.22	.30	.30
**13**	.53	.42	.50	.31	.34	.28	.34	.41	.38	.36	.19	.41	**.64**	.29	.53	.45	.25	.31	.36	.38
**14**	.34	.47	.32	.26	.45	.34	.45	.37	.39	.42	.29	.35	.29	**.77**	.42	.45	.50	.32	.48	.55
**15**	.69	.70	.46	.32	.69	.54	.61	.39	.50	.56	.39	.30	.53	.42	**.73**	.64	.44	.36	.32	.60
**16**	.39	.70	.33	.24	.61	.45	.58	.27	.55	.42	.33	.30	.45	.45	.64	**.90**	.42	.30	.27	.61
**17**	.39	.50	.39	.31	.44	.36	.50	.42	.39	.47	.31	.28	.25	.50	.44	.42	**.75**	.39	.43	.47
**18**	.33	.27	.57	.63	.45	.47	.47	.63	.37	.33	.47	.22	.31	.32	.36	.30	.39	**.79**	.30	.47
**19**	.39	.45	.32	.25	.23	.34	.27	.25	.43	.34	.25	.30	.36	.48	.32	.27	.43	.30	**.77**	.36
**20**	.58	.70	.33	.33	.77	.37	.57	.37	.63	.28	.33	.30	.38	.55	.60	.61	.47	.47	.36	**.79**

For each cluster, the DRP with the highest average native overlap value to all other DRPs in the cluster was selected as the representative member to be used as the basis for phage display libraries (the calculation of native overlap is described in Methods). These average native overlap values for the representative DRPs are displayed along the matrix diagonal in bold. Additionally, pairwise structural alignments of all representatives were computed with SALIGN; the resulting native overlap values are displayed in off-diagonal cells in the matrix

**Table 2:** Summary of clusters

Cluster	Name	# Members	Avg Seq Id	Avg Length	Scaffold
1	Knottin CSαβ I	115	21.8	38	2crdA
2	Knottin Classic ICK	98	23.4	24	2jtbA
3	Insulin	58	42.4	23	3gkyC
4	Small Conotoxin	52	24.9	12	1e76A
5	Knottin Cyclotide ICK	48	30.9	30	3e4hA
6	Small Hairpin	42	15.9	16	1wo0A
7	EGF-like Hairpins	39	17.0	19	2oqjL
8	Medium Conotoxin	35	21.0	17	2uz6K
9	α-Defensin	30	53.4	31	3lo2B
10	β-Defensin	25	51.1	37	2nlsA
11	Large Hairpin	22	21.5	17	1gm2A
12	Crambin	19	56.8	46	1orlA
13	Helix-Loop-Helix	19	12.2	34	1bzbA
14	LDL Receptor	17	30.4	39	2kriB
15	Knottin CSαβ II	12	19.7	29	1du9A
16	PMP Inhibitor	11	59.0	35	2f91B
17	TNF Receptor	11	42.6	39	1xu2R
18	Large Conotoxin	10	24.6	19	1tckA
19	Tryptase Inhibitor	10	42.2	39	2kmoA
20	Anti-microbial Peptide	9	49.2	30	1mmcA

Clusters are sorted by number of members. Name: Manually assigned name, derived from the most frequent SCOP fold assignment for each cluster. Avg Seq Id: Average pairwise sequence identity of all DRPs in the cluster. Avg Length: Average sequence length of all DRPs in the cluster, derived from the sequence resolved in the PDB structure. Scaffold: Selected representative for the cluster

### Sequence dissimilarity of peptides in the same cluster

To examine the relationship between structure and sequence in the clusters, the average pairwise sequence identity among all members in each cluster was calculated (Table 2). No cluster in the top 20 had average sequence identity higher than 60%, and ten of the clusters had average sequence identity of less than 25%, indicating that the binding partners of DRPs within the same cluster were likely diverse. Sequence conservation was visualized by coloring the DRP structures according to the degree of conservation at each residue position (Fig. 4). In most of the clusters, the yellow conserved areas consisted of residues surrounding the disulfide bonds which likely contribute to DRP stability, while the blue diverse areas were generally found in the loops and surface regions, which are more likely to interact with other proteins and are viable candidate regions for randomization through phage display.

### Panning against human interleukin-23

To demonstrate the utility of our clustering approach, phage display libraries were constructed for three representative DRPs and panned against the human cytokine protein interleukin-23 (IL-23). Inhibiting IL-23 binding to its receptor (IL-23R) reduces inflammation and other adverse immune responses; thus, IL-23 is an attractive therapeutic target, particularly in non-responders to anti-TNF agents [42]. The interaction between IL-23 and IL-23R is a typical protein-protein interaction involving a large flat recognition surface, and a low molecular weight binder to IL-23, which would prevent complex formation, would be challenging to discover.

**Fig. 5:**
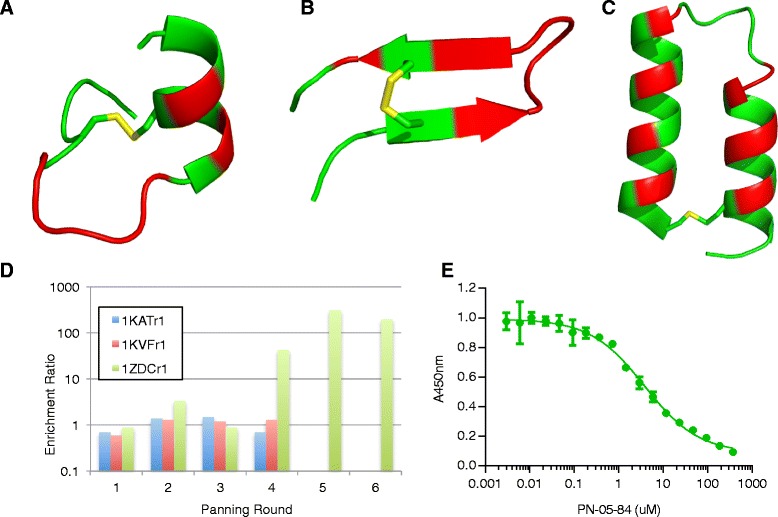
Phage display experiment. **a** Structure of the peptide scaffold for phage library 1KATr1. Variable residue positions are colored *red*, and disulfide bonds in *yellow*. The same representation is used for the 1KVFr1 (**b**) and 1ZDCr1 (**c**) library scaffolds. **d** Enrichment ratios across successive rounds of phage panning for the three libraries. Panning was discontinued after the fourth round for 1KATr1 and 1KVFr1 due to a lack of enrichment. **e** Standard curve resulting from competition ELISA experiment to assess inhibition of IL-23/IL-23R complex formation by the PN-05-84 clone

The three selected DRPs were (1) from the large conotoxin cluster, an antagonist of vascular endothelial growth factor (PDB identifier 1KAT [43]); (2) from the small hairpin cluster, an agonist of erythropoietin (1KVF [44]), and (3) from the helix-loop-helix cluster, a derivative of Protein-A (1ZDC [45]). These DRPs were chosen due to their different secondary structure classes (loops, sheet, helix respectively), which may maximize their binding diversity. Moreover, the first two peptides were themselves products of phage display libraries, which suggested experimental tractability. One library was designed for each DRP scaffold; these libraries were referred to by the PDB identifiers of their scaffolds (1KATr1, 1KVFr1, and 1ZDCr1; full library design described in Fig. 5a-c and Additional file 1: Table S4.

The libraries were constructed and screened against immobilized IL-23 in successive rounds of panning. Enrichment ratios, which compare the output titers in the target selection to a background negative control, were determined after each round. Panning the 1KATr1 and 1KVFr1 libraries was halted after four rounds due to the lack of enrichment; however, the 1ZDCr1 library showed significant enrichment after the fourth round and was thus subjected to two further rounds of panning (Fig. 5d). After the sixth round, individual clones were isolated and assessed for binding with a phage ELISA. Positive clones were sequenced. One sequence, PN-05-84 was synthesized and tested in a competition ELISA to assess inhibition of IL-23 binding to IL-23R. PN-05-84 inhibited binding with an IC_50_ of 3.3 μM (Fig. 5e); it is likely that this binding potency could be improved through medicinal chemistry approaches, as we have done previously for other targets [36].

## Discussion

### Advantages of using structurally diverse scaffolds in phage display

The utility of phage display for developing lead compounds is well appreciated, with its proficiency deriving from the ability to make and screen libraries of up to 10^12^ sequences and the linkage of genotype to binding phenotype [46]. Typical peptide phage display involves the creation of large libraries sampling enormous sequentially continuous sequence space on unstructured peptides that assume structure only upon binding the target bait. The disordered nature of these peptides weakens the utility of phage display, as in some instances it is impossible to select the weakly active unstructured peptides from the vast majority of inactive peptides.

On the other hand, phage display of DRPs allows for sampling different sequences on a discontinuous surface in conformationally controlled structure space. One of the key requirements in discovering leads, and ultimately drugs, is to present the required functional groups in a sufficient orientation to yield potent and selective molecules at the target of interest, while optimizing the desired drug-like physicochemical features. This requirement is achieved through the common discontinuous surface patches of DRPs, described here, which represent naturally occurring fractions of chemical structure space explored by nature, and as such are biologically relevant. Consequently, the probability of obtaining hits may be higher than with unstructured peptide phage libraries, or with small molecule scaffold topologies explored in combinatorial chemistry, which are typically not biologically relevant. This probability further increases when multiple structurally distinct libraries are panned. To develop such libraries, we require a set of diverse DRP scaffolds.

These scaffolds were identified by the protocol in this study, which clusters DRPs by structural similarity over their full length and refines some of the clusters by incorporating the structural conservation of disulfide bonds and by resolving artifacts of the hierarchical clustering method through reassigning singletons to more populated clusters. The result was an experimentally tractable set of 20 structurally diverse, representative scaffolds from the most populated clusters that could be used for constructing phage display libraries.

DRPs are an emerging source of lead compounds in drug discovery due to their inherent chemical and biological stability characteristics, as exemplified by the marketed orally delivered drug linaclotide [31]. DRP phage display libraries may provide a valuable, generic resource for the discovery of additional DRP modulators of protein-protein interactions and may help alleviate the low hit rate currently plaguing the pharmaceutical sector.

Next, we discuss the clusters in the context of DRP properties as well as the phage display process.

### Evolutionary insights obtained through DRP clustering

Clustering DRPs by structure, and incorporating no annotation other than previous identification of some DRPs as knottins, revealed insights into DRP structure. Many clusters contained DRPs possessing structurally conserved disulfide bonds, as demonstrated through qualitative visual analysis. This conservation tends to occur in clusters with larger folds; for example, the EGF-like hairpin, α-defensin, crambin, and TNF receptor clusters all had near-complete conservation of disulfide bonds (Fig. 4). Unlike the knottins, these clusters were aligned over the full length of their sequences in the initial hierarchical clustering step (Fig. 1a, step ii), yet resulted in high structural overlap among equivalent disulfide bonds, suggesting that there is strong selective pressure to maintain these cysteine pairings. This result corroborated the findings of a previous study that showed high disulfide bond conservation within the SCOP “Small Peptide” fold class [47].

The clusters with the least amount of disulfide bond conservation were those containing peptides with shorter sequence lengths; examples include Small Hairpin and Small Conotoxin clusters. The N and C termini are proximal to each other in both of these folds, and in these peptides there were a number of possible position pairs between which disulfide bonding was sufficient to maintain the structure. Thus, there was less evolutionary pressure to conserve a disulfide bond between specific positions than there is in longer DRPs. Furthermore, in many cases there was likely no evolutionary pressure at all, as these clusters included peptides with diverse functions, or were engineered. Nevertheless, cluster members assume similar folds, likely due to the reduced conformational sampling available to peptides of this size possessing two or more cysteines.

In addition to their utility as phage display libraries, and insight into disulfide bond conservation, the clusters allow for a broader view of DRP evolution. For example, we wondered how DRPs from different species were distributed across the clusters. In a simple analysis, each PDB structure was mapped to its Uniprot accession [48] to obtain its annotated species, and the total number of species, as well as the ratio of DRPs to unique species in each cluster, was calculated (Additional file 1: Table S5). Most clusters were composed of DRPs expressed across a number of different species. For example, the EGF-hairpin cluster contained 34 peptides from 21 species; the average ratio of DRPs per species across the top 20 clusters was 2.85. This result demonstrates the broad phylogenetic distribution of a small number of DRP folds. These ratios were also calculated across taxonomic classification at higher levels. Some clusters were distributed broadly even at the kingdom level; both the Knottin CSαβ I and Classic ICK clusters included DRPs in three kingdoms (Metazoa, Viridiplantae, and Fungi, using Uniprot assignments). As it is unlikely that a single fold resulted from three or more convergent events, this distribution across kingdoms suggests that these scaffold forms may have emerged early in the course of eukaryotic development, as has been hypothesized previously [12, 49]. Other clusters imply a narrower evolutionary path; for example, the Crambin cluster comprised DRPs from only one class, Liliopsida, which represents herbaceous monocots such as common wheat and barley.

### Discussion of selected cluster folds

Detailed analysis of certain clusters may elucidate structure-function relationships among these unique peptides. First, the top 20 clusters included 4 composed primarily of knottin folds. Knottins are characterized by a cysteine-knot architecture; generally, these peptides possess a C-terminal β-sheet connected to the N-terminal region by two or three disulfide bonds [10]. Loops in knottins had high structural variability, rendering these peptides problematic when clustering them by native overlap over the full sequence. Thus, an intermediate step in the protocol reclustered knottins based on structural overlap across their core disulfide bonds, which allowed for selection of a scaffold that was similar in core structure to other members of its cluster, but had the potential to present a binding surface in a similar conformation to a large number of other knottins, particularly if the loop size were to be varied as part of the phage display experiment. Knottin disulfide bonds exhibited a remarkable degree of structural overlap, with 229 DRPs grouped into only 4 clusters (Additional file 1: Table S2).

Notably, each of these clusters generally included peptides assuming a single knottin fold. Most of the peptides in the largest cluster were of the CSαβ fold, in which the N-terminal region forms an α-helix containing two or three cysteines that disulfide-bond with partner cysteines in the C-terminal β-sheet. The regular spacing of the cysteines along the α-helix leads to the observed tight clustering of peptides according to structurally equivalent disulfide bonds. Additional CSαβ DRPs were present in cluster 15. Cluster 2 consisted of peptides assuming the classic ICK fold, with a few cyclotide ICKs, while cluster 5 was composed of almost all cyclotide peptides. These two knottin forms can be differentiated by the spacing of their six cysteines along the peptide sequence; classic ICKs have an arbitrary number of residues between the fourth and fifth cysteines, whereas cyclotides have only a single residue in this region. These cysteine spacing differences contributed to these folds being assigned separate clusters according to disulfide structural equivalency.

Knottins in different clusters generally had different functions. The Knottin CSαβ I cluster was the largest of all DRP clusters, with 115 members. Of these peptides, 49 were potassium channel inhibitors, drawn from 15 species; 17 were defensins; and 12 assumed an EGF-like fold, nine of which were found in human coagulation factors (Additional file 1: Table S3). None of these functions was assigned to DRPs in the Knottin Classic ICK or Knottin Cyclotide ICK clusters (although five more potassium channel inhibitors were present in Knottin CSαβ II). Instead, the Classic ICK cluster was composed of a diverse array of toxins, including conotoxins, agatoxins, and theraphotoxins, while the Cyclotide ICK cluster included trypsin inhibitors and cyclotides with antimicrobial functions, predominantly from plants. Thus, core disulfide bond equivalency appeared to correlate strongly with different functions mediated by surface loops across different knottin folds.

Similar to knottins, hairpin peptides fell into multiple clusters: Small Hairpin (averaging 14.3 residues in length) and Large Hairpin (averaging 21.6 residues). Despite these peptides all consisting of simple β-strand pairs joined by one or two disulfide bonds, multiple clusters were created due to the significant differences in sequence lengths, similar to knottins. The Large Hairpin cluster afforded more space along the sequence to incorporate disulfide bonds; peptides in this cluster averaged 1.59 disulfide bonds, compared with 1.22 in the Small Hairpin cluster. Additionally, Large Hairpins were more likely to be found in nature; 70% of cluster members were fully expressed peptides or isolated as a fragment from a full protein. Nearly all of these peptides were serine protease inhibitors or membrane pore-forming peptides that exhibit antibacterial and antiviral activity. On the contrary, 72% of Small Hairpins were engineered, for example as the products of phage display libraries or synthesized to examine how different amino acid residue types contribute to the β-hairpin fold (Additional file 1: Table S3).

Many hairpins had disulfide bonding patterns similar to those of the members of the Small Conotoxin cluster (Additional file 1: Table S6). For example, there were two members of the Small Hairpin cluster and seven members of the Small Conotoxin cluster with a CX_9_C motif, where X is any amino acid residue type other than cysteine; in fact, this motif represented the full sequence for the engineered DRPs 1n0aA in the Small Hairpin cluster and 3p72B in the Small Conotoxin cluster. This result indicates that care must be taken in designing phage libraries to ensure that a scaffold based on a hairpin maintains its fold; for example, certain amino acid residue types that confer hairpin properties should not be varied. Alternatively, hairpins with fewer residues between bonded cysteines could have their full surface varied with the NNK codon, which would resemble traditional random peptide libraries that have been the focus of earlier studies [44].

### Likelihood of additional uncharacterized DRP folds

We have shown that 85% of DRPs with solved structures fall into 20 fold classes, although these 20 folds only represent approximately 1/4 of all known DRP folds given that 81 clusters were created overall. Thus, DRP sequences are distributed non-uniformly across known DRP folds, as has been observed with globular and membrane protein sequences in general [50]. However, the question remained whether the PDB is biased toward DRPs with certain fold classes. The initial filtering step in our protocol was intended to reduce any bias by removing redundant proteins (Fig. 1a, step i). Filtering with the 90% sequence identity threshold (instead of 100%) still resulted in 79.3% of DRPs falling in the top 20 clusters (data not shown), suggesting that the non-uniform size of the DRP clusters was not an artifact of our procedure or the DRP sample in the PDB. Notably, among the 81 clusters output by our pipeline, 43 contained only one member, suggesting that there are additional unknown folds that are assumed by a small number of DRP sequences.

### Phage display application

We have identified 20 structurally distinct peptides that can be used as scaffolds for phage display. The likelihood of success in a phage display experiment is dependent on library design. DRP scaffolds offer unique challenges and opportunities in this respect. The most important design consideration is the choice of residues to vary. We propose that these residues should be located in regions that are not conserved in sequence, to decrease the probability of affecting peptide folding kinetics and stability. To this end, the degree of sequence conservation was quantified across equivalent residue positions within a cluster; the blue non-conserved regions in Fig. 4 suggest optimal surfaces to vary in phage libraries. These regions frequently occur on loops and are solvent-exposed; additionally, there is only one such surface on many of the selected cluster representatives, which results in a limited number of residue positions from which to choose for variation. Finally, if the natural binding surface of the DRP is known, the residues in this region should also be considered as candidates to vary through phage display as has been done previously [40].

Additionally, while we have identified a set of diverse scaffolds, the utility of our protocol increases if the varied surfaces themselves are structurally diverse as well. This property is illustrated in Fig. 5a-c, where the selected surfaces on particular DRPs are composed of different combinations of secondary structures, including loops, helices, and sheets. We suggest these varied surfaces would be diverse across scaffolds even if surfaces were selected randomly on each DRP; there is little structural overlap across the full length of the scaffolds, and thus there is likely to be little overlap across subsets of the scaffolds. An exception is the α- and β-defensins, where the β-class includes an N-terminal helix not present in the α-class, with the remainder of the peptide chains being structurally similar (Fig. 4; clusters 9–10). Thus, the β-defensin varied surface could include this helix to ensure it is structurally distinct from the α-defensin surface.

These considerations were applied to design three phage libraries based on selected cluster representatives. Different secondary structures were accounted for, and regions from discontinuous surfaces were varied to increase the binding footprint (Fig. 5). No enrichment was observed from panning the 1KATr1 and 1KVFr1 libraries against IL-23. This result demonstrates the drawback of relying on a single phage library to produce hits using a generic panning strategy. It is likely that none of the sequences produced through phage variation had structural complementarity to IL-23 and the phage library would thus not produce a positive result regardless of the sequence diversity sampled. In contrast, panning 1ZDCr1 resulted in a modestly potent 3 μM hit. Thus, even though the theoretical sequence diversity is similar across the three libraries, only 1ZDCr1 yielded hits in a generic panning strategy, which illustrates the value of presenting sequence diversity in different topological shapes, particularly in those that confer the favorable chemical and biological stability of DRPs.

## Conclusions

We have developed an automated protocol for clustering DRPs and applied it to group 1,411 peptides into 81 clusters, with 85% of those DRPs falling into only 20 most populous clusters. Given the likelihood that diverse DRP sequences assume a limited number of folds, similar to proteins as a whole, these 20 clusters appear to reflect the structure and function of the majority of DRPs found throughout nature. Constructing phage libraries comprising 10^10^ sequences displayed in topologically distinct conformations (Fig. 4) and panning these libraries could result in binders that disrupt protein-protein interactions associated with disease. Collectively, these libraries sample immense chemical space displayed in well-defined discontinuous surfaces that are composed of distinct combinations of secondary structures. By binding to flat protein interfaces, peptides derived from these libraries represent a promising alternative to the traditional monoclonal antibody approaches, particularly when considering their non-immunogenic character [15], protease stability [23] and potential for oral delivery [24]. The usefulness of our approach has been demonstrated by the identification of a μM binder from the initial panning of phage libraries based on only three scaffolds against the IL-23 target.

## Methods

### Definition of core terms

Prior to describing the full protocol used in this study, we first define the distance metrics “Native overlap” and “Disulfide distance”, and give a generic description of the hierarchical clustering procedure.

### Native overlap

Native overlap was defined as the fraction of Cα atoms in one DRP that were within 3.5 Å of the corresponding atoms in a second DRP following structural alignment of the first DRP to the second DRP. Thus, a native overlap of 1.0 meant that all equivalent residues across the aligned DRPs are within 3.5 Å of each other and there are no gaps in the alignment (i.e. every residue in one DRP had an equivalent in the other). Structural alignments were performed using the iterative_structure_align() command in MODELLER version 9.10 [51]; this command implemented the SALIGN algorithm [52].

### Disulfide distance

To align by disulfide bonds equivalent across a DRP pair, the structurally equivalent disulfide bonds were first identified. This identification first enumerated all possible mappings of disulfide bonds from the first DRP to the second. Additionally, for each mapping, it was unknown which cysteines were equivalent in an equivalent disulfide bond; therefore, all possible cysteine equivalencies are generated. Thus, if two DRPs each had three disulfide bonds, there were a total of 48 mappings enumerated (six disulfide bond mappings and eight possible cysteine equivalencies for each). Then, for each mapping, a structural alignment was performed through a least-squares superposition of the mapped cysteine Cα atoms. Following the superposition, the sum of the three-dimensional distances between all equivalent Cα atoms as well as all equivalent Sγ atoms was taken as the disulfide distance for that mapping. This procedure was repeated for all mappings; the final mapping was the one with the smallest disulfide distance. If the two DRPs had a different number of disulfide bonds, then each mapping had an unmapped disulfide bond, which was not considered in the sum of equivalent distances.

### Average-linkage hierarchical clustering

A canonical bottom-up, average-linkage hierarchical clustering procedure was implemented to cluster the DRPs. This procedure has been extensively described [53]. Briefly, each DRP was initialized as its own cluster, and the distances between all cluster pairs were calculated (native overlap for the initial clustering and the disulfide distance for knottin reclustering). The two clusters with the shortest average distance were merged, and the average distances between the merged cluster and all other clusters were recalculated. ‘Average linkage’ refers to calculating the average distance of all pairs of DRPs across a pair of clusters. The procedure iterated, with each step consisting of merging the pair of clusters with the shortest average distance and recalculating all distances. The iteration terminated when the shortest average distance is below some cutoff; all subtrees in the cluster hierarchy that are rooted below this cutoff were the output clusters of the algorithm (Fig. 1b).

### Overview of protocol

The PDB was searched for all protein chains with fewer than 50 amino acid residues and between one and four annotated disulfide bonds. Pairwise structural alignments of all such DRPs were computed using the SALIGN algorithm. The output of these alignments were first used to filter identical DRPs from the dataset; any DRP that had 100% sequence identity and 1.0 native overlap to another DRP was discarded. The result was the initial set of filtered DRPs that were used as input to the main pipeline (Fig. 1a, step i).

Next, the filtered DRPs were grouped using the hierarchical clustering algorithm, using native overlap as the distance metric with a cutoff of 0.7 (Fig. 1a, step ii). This cutoff was selected manually through visualization of the resulting clusters; alternate cutoffs of 0.6 and 0.8 were also assessed and rejected. Any cluster containing four or more peptides annotated with the SCOP “knottin” fold (SCOP identifier g.3) were considered “knottin clusters”; peptides from these clusters were pooled and reclustered hierarchically, using the disulfide distance metric and imposing a cutoff of 2.0 Å. Here again, the cutoff was determined through visualization of the resulting clusters, with the cutoffs of 1.5 Å and 2.5 Å also being considered, but rejected (Fig. 1a, step iii). Together with all non-knottin clusters from the initial clustering step, these reclustered knottin clusters formed a set of intermediate clusters. These intermediate clusters were used as input to singleton post-processing steps.

### Singleton reclustering

The intermediate clusters included a number of DRPs that didn’t fall into one of the 25 most populated clusters, but still had significant structural similarity to a DRP that did fall in such a cluster. Such a DRP was referred to as a ‘singleton’ for these purposes; the number 25 was chosen as a cutoff point because the number of DRPs per cluster decreases significantly for the 26^th^ most populous cluster (Fig. 3). A singleton was defined as any DRP ***x*** from a cluster not ranked in the top 25 clusters by size where there existed another cluster ***I*** that fulfilled two conditions: (1) ***I*** was ranked in the top 25 clusters by size and (2) ***I*** contained a reference DRP ***y*** that aligned to ***x*** at a native overlap above the cutoff used in the initial hierarchical clustering process. When these conditions were met, ***x*** was removed from its original cluster and added to ***I***. This procedure was repeated twice. The first iteration used the length of the longer DRP in the denominator when calculating the native overlap, which was the same procedure used in the initial hierarchical clustering step. These singletons were referred to as ‘longer singletons’ (Fig. 1a, step iv). The resulting clusters were reranked by size and the top 25 were considered as new instances of ***I*** as above. Then, new singletons were identified in the less populated clusters, this time considering the length of the shorter DRP in the native overlap calculation. These peptides, denoted ‘shorter singletons’, were reassigned to the larger clusters, resulting in the final output of the protocol (Fig. 1a, step v).

### Selection of representative DRPs

For each of the top 20 clusters, the average native overlap value between each DRP and all other DRPs in the cluster was calculated. The peptide that had the largest average native overlap value was selected as the representative for that cluster.

### Sequence identity calculation

For each cluster, sequence identities were calculated for all DRP pairs. For each DRP pair, the structural alignment computed by SALIGN was used to identify the structurally equivalent residues across the two DRPs. The sequence identity was calculated by dividing the number of equivalent residues having the same amino acid residue type by the number of residues in the full sequence of the longer DRP. The average sequence identity for the cluster was the average of sequence identities for the DRP pairs in the cluster.

### Visualization of structural alignment and sequence similarity

For each cluster, a multiple structure alignment was performed for all DRPs using SALIGN. A multiple sequence alignment was produced based on the structure alignment and used as input to the program AL2CO [54], which quantified the overall degree of conservation at each position in an alignment. The ‘sum of pairs’ method of AL2CO was used, using the BLOSUM62 scoring matrix [55] to compare similar amino acid residue types. AL2CO calculated normalized scores at each position ranging from −2 to 2; these scores were scaled to RGB color values that could be used by the structure visualization program PyMol [56] to color individual residues; thus, each residue was colored on a RGB scale of blue [0, 0, 255] to yellow [255, 255, 0]. Commands to perform the coloring were automatically generated and saved in a PyMol script, which read the aligned structures generated by SALIGN and colored each residue for each DRP according to the degree of sequence conservation in the alignment.

### Phagemid libraries

All libraries used in phage selection were phagemid based, containing an arabinose promoter driving the expression of fusion proteins of the following form: an STII secretion signal, followed by a hemagglutinin tag, a four residue linker sequence, the peptide library, another four residue linker sequence, and the M13 gene-3 coat protein. The peptide libraries were amplified using oligonucleotides containing the variable positions encoded by NNK codons. The DNA fragments encoding the desired scaffolds were then cloned into the phagemid vector and transformed into electrocompetent *E. coli* XL1-Blue cells.

### Selection of IL-23 binding peptides from naive peptide phage libraries

For library selection, IL-23 recombinant protein was immobilized on a biotinylated anti-p40 antibody (eBiosciences, C8.6, #13-7129-81) conjugated to Dynabeads® MyOne™ Streptavidin T1 (Life Technologies # 65601). Approximately 1×10^12^ phage particles in PBS containing 1% BSA were added to the beads with or without immobilized IL-23 protein and incubated for 1 h at room temperature. Unbound phage particles were removed by washing the beads with PBS containing 0.05% Tween 20 (PBST). Bound phage particles were eluted from the beads with 100 mM TEA, incubated for 10 min at room temperature, followed by immediate neutralization with Tris base. The eluted phage particles were amplified by infecting log phase XL1-Blue. After shaking for 2 h at 37 °C, the cultures were superinfected with M13KO7 helper phage and grown for another 2 h at 37 °C. Kanamycin was added to a final concentration of 70 μg/mL, and the cultures were grown overnight at 30 °C. Phage particles were harvested by first incubating the supernatant with 20% PEG 8000/NaCl solution (Teknova #P4138) for 30 min on ice, followed by centrifugation. The phage pellet was suspended in PBS containing 1% BSA and sterile filtered through a 0.2 μM PES filter unit. The amplified phage pool was then incubated with the immobilized target, washed, eluted and amplified as above for another 3 to 5 rounds. To ensure specific binding, all amplified phage pools were pre-incubated with biotinylated anti-IL-23p40 antibody conjugated to Dynabeads® MyOne™ Streptavidin T1 prior to the addition of the target. A successful selection requires a high enrichment ratio for target specific phage clones. The enrichment ratio was calculated by dividing the number of phage particles recovered in the presence of IL-23 by that in the absence of IL-23.

Individual clones from round 6 were analyzed by single-point phage ELISAs. Positive monovalent phage clones were identified as those that bound the antibody captured IL-23 and not the antibody. Positive clones were subjected to DNA sequencing.

### Phage ELISA

To facilitate the rapid analysis of phage clones, 96 well formats for phage growth and ELISAs were used. Individual XL-1 Blue colonies harboring phagemid were picked into Growth Media (2X YT supplemented with antibiotics) in a deep 96 well plate. After overnight growth, cultures were diluted 1:20 into fresh Growth Media and grown at 37 °C until OD600 reached 0.6. Cultures were superinfected with M13KO7 helper phage and grown for another 2 h at 37 °C. Kanamycin was added to a final concentration of 70 μg/ml, and the cultures were grown overnight at 30 °C. Phage supernatants were collected by centrifugation, transferred to fresh 96 well plates and used directly in single-point phage ELISA.

For phage ELISA, a 96 well Immulon® 4HBX plate (VWR #62402-959) was coated with 400 ng/well of streptavidin and incubated overnight at 4 °C. The wells were washed two times with PBST, blocked with PBS containing 1% casein for 1 h at room temperature, and washed again three times with PBST. A biotinylated anti-p40 antibody was added to each well at 250 ng/well diluted in Assay Buffer (PBS containing 0.5% casein), washed three times with PBST, followed by addition of Assay Buffer in the presence of absence of IL-23 at 50 ng/well. The plate was washed three times with PBST. Phage supernatants were added to individual wells and incubated for 1 h at room temperature. The plate was then washed four times with PBST. The presence of phage particles was detected by incubation with a horseradish peroxidase (HRP) conjugated anti-M13 antibody (GE Healthcare #27942101) diluted 1:5000 in PBS for 1 h at room temperature. Finally, the plate was washed three times with PBST. Signals were visualized with TMB One Component HRP Membrane Substrate (SurModics #TMBW-1000-01), quenched with 2 M sulfuric acid and read spectrophotometrically at 450 nm.

### Peptide synthesis

Peptides were synthesized using the Merrifield solid phase synthesis techniques on a 12 channel multiplex Symphony® peptide synthesizer (Protein Technologies, Inc.) and were assembled using O-Benzotriazole-N,N,N’,N’-tetramethyluroniumhexafluorophosphate (HBTU) and N,N-diisopropylethylamine (DIPEA) coupling conditions. Rink Amide MBHA resin was used for peptides with C-terminal amides and pre-loaded Wang Resin with N-α-Fmoc protected amino acids was used for peptides with C-terminal acids. The coupling reagents (HBTU and DIPEA premixed) and amino acid solutions were prepared in dimethylformamide (DMF) at a concentration of 100 mM. The peptides were assembled using standard Symphony® protocols. Pre-loaded Wang resin (250 mg, 0.14 mmol, 0.56 mmol/g loading, 100–200 mesh) or MBHA resin (250 mg, 0.15 mmol, 0.6 mmol/g loading, 100–200 mesh) was placed in each reaction vial and washed twice with 4 mL of DMF followed by 2 x 10 min treatments with 2.5 mL of 20% 4-methylpiperidine/DMF (conditions for Fmoc deprotection). Either the Wang resin or the Rink Amide MBHA resin was then washed three times with DMF (4 mL), followed by addition of 2.5 mL of amino acid and 2.5 mL of a HBTU-DIPEA mixture. After 45 min of reaction with frequent agitation, the resin was filtered and washed three times with DMF (4 mL). This process was then repeated.

The coupling reaction was carried out twice for the first 25 amino acids and three times for the remaining amino acids. The assembled peptide on resin was then cleaved using a 2 h treatment with cocktail reagent K [57]. The cleaved peptides were precipitated in cold (0 °C) diethyl ether, followed by washing two times with diethyl ether and air drying. The crude peptides were then submitted to an oxidation reaction in order to form the disulfide bridge. The crude peptide was dissolved in 50% acetonitrile/water at a concentration of 0.5 mg/mL. A saturated solution of iodine in methanol was added dropwise until a yellow color persisted. Excess iodine was quenched by the addition of solid ascorbic acid until the solution became colorless. The resulting solution was purified by preparative reverse-phase HPLC: Phenomenex® Luna C18 column (10 μm, 300 Å, 250 × 21.2 mm) using buffer A (0.1% trifluoracetic acid (TFA) in water), buffer B (0.1% TFA in acetonitrile) gradient 33–55% buffer B over 45 min, flow rate 20 mL/min, detection at 220 nm. Fractions containing the desired product were pooled and lyophilized to give a white solid. The amino acid sequence of the test peptide PN-05-84 was FNMQCLRRMSEAGVDPNLNQEQRWAKIKSIMDDC.

### IL23-IL23R competitive binding ELISA

Immulon® 4HBX plate was coated with 200 ng/well of IL23R_huFC and incubated overnight at 4 °C. The wells were washed three times with PBST, blocked with PBS containing 5% PhosphoBLOCKER (Cell Biolabs #AKR-103) for 1 h at room temperature, and washed again three times with PBST. Serial dilutions of test peptides and IL-23 at a final concentration of 0.9 nM in PBS were added to each well, and incubated for 2 h at room temperature. After the wells were washed, bound IL-23 was detected by incubation with 50 ng/well of goat anti-p40 polyclonal antibodies (R&D Systems #AF309) diluted in PBS for 1 h at room temperature. The wells were again washed four times with PBST. The secondary antibodies, HRP conjugated donkey anti-goat IgG (Jackson ImmunoResearch Laboratories #705-035-147) diluted 1:5000 in PBS was then added, and incubated for 30 min at room temperature. The plate was finally washed as above. Signals were visualized with TMB One Component HRP Membrane Substrate, quenched with 2 M sulfuric acid and read spectrophotometrically at 450 nm.

### Software availability

The Python scripts that were used to run most of the computational components of these methods, including building the distance matrices, running the clustering pipeline, and performing multiple structure alignments of DRPs within each cluster for visualization purposes, are available in Additional file 2.

## Additional files

Additional file 1: Contains six supplemental tables in separate tabs of a Microsoft Excel Workbook. Legends for each table are included in the “Table Summary” tab of this file. (XLSX 106 kb)
Additional file 2: Python scripts required to run the method, as well as a PDF describing usage and requirements. (ZIP 89 kb)

## Abbreviations

DRP: Disulfide-Rich Peptide
IL-23: Interleukin-23
IL-23R: Interleukin-23 Receptor
IL-6: Interleukin-6
PDB: Protein Data Bank
SCOP: Structural Classification of Proteins
TNF: Tumor Necrosis Factor
