# Supplementary material for: Clustering of disulfide-rich peptides provides scaffolds for hit discovery by phage display: application to interleukin-23
Source: BMC Bioinformatics. 2016 Nov 23;17:481. doi: 10.1186/s12859-016-1350-9 (PMC5120537; doi:10.1186/s12859-016-1350-9)
Supplement: Additional file 2: — Python scripts required to run the method, as well as a PDF describing usage and requirements. (ZIP 89 kb) [file 12859_2016_1350_MOESM2_ESM.zip › DRP_Clustering_Usage.pdf]

REQUIREMENTS

- This code has only been tested on Python 2.7.12. It will not work with Python 3.
- MODELLER must be installed and in your PYTHONPATH.
- All python modules included in this supplemental file must be in your PYTHONPATH
- A local mirror of the PDB must be available; see below

OVERVIEW

There are four overall steps in running the pipeline:

1. Preparing the PDB coordinate files
2. Creating distance matrices both for the native overlap and disulfide bond metrics
3. Running the cluster pipeline
4. Running the visualization step that aligns PDB files in the final clusters.

Note: Commands are specified with '>'; everything else is descriptive text. Commands listed do not include the full path to the python module or any input data files but these must be specified if not in CWD.

TERMINOLOGY

DRP Code: a 5 character code representing a single chain DRP PDB structure. The first 4 characters are the PDB ID and the fifth is the chain. For example, 2ny9X refers to PDB ID 2ny9 chain X (a defensin protein).

DRP Query File: a .txt file specifying DRPs that will be considered in your pipeline run. You must specify this input set yourself. The list of DRPs in our publication is a potential starting point, but you may wish to include more or fewer. The DRP query file should have one DRP Code per line. In the examples given, this file is named drpQueryFile.txt

PDB Directory: This is the location of your PDB files. It is expected to be a mirror of the full PDB FTP site and contain a number of two letter subdirectories representing PDB's divided directory structure (i.e., the same structure as ftp://ftp.wwpdb.org/pub/pdb/data/structures/divided/pdb/). Note that PDB files in here follow the form 'pdb2ny9.ent.gz'. In step 1, coordinate files are copied from here and stripped down to only retain the chain in the DRP code and the SSBOND connectivity annotation. In the examples given, this directory is named dividedPdbDir.

STEP 1: Preparing the PDB Coordinate files

```
>mkdir localPdbDir
>cd localPdbDir
>python setupPdb.py -q drpQueryFile.txt -p dividedPdbDir
>cd ..
```

This copies PDB files into localPdbDir. This directory can obviously be any path, but this step outputs to CWD. Downstream steps will refer to this path, which we will just call localPdbDir

This step also creates a file named drp_lengths.txt which is needed in step 3.

STEP 2: Create distance matrices

```
>python pairwiseAlign.py -q drpQueryFile.txt -o nativeOverlapOutput.txt -m full_drp -p localPdbDir
>python pairwiseAlign.py -q drpQueryFile.txt -o disulfideOutput.txt -m disulfides -p localPdbDir
>grep longer_fraction nativeOverlapOutput.txt > longerFraction.txt
>grep longer_sequence_product nativeOverlapOutput.txt > similarityProduct.txt
>grep shorter_fraction nativeOverlapOutput.txt > shorterFraction.txt
```

Note:

- The first two commands will take a while depending on the size of your input (order of O(n^2); each pairwise comparison takes ~1s so >1000 DRPs will take >130h). Alternatively this step could be distributed on an SGE cluster by running the individual alignment scripts alignNativeOverlap.py and alignDisulfides.py, which each take two DRP codes as input (pairwiseAlign.py is just a wrapper around those two alignment scripts that runs them for all pairs in drpQueryFile.txt). Output for all SGE cluster runs of alignNativeOverlap.py would need to be concatenated prior to Step 3; the same is true for all SGE cluster runs of alignDisulfides.py (as opposed to pairwiseAlign.py which outputs everything to the same file). See usage for those two scripts.

- For this step, if the PDB file is not found, it won't stop the full pairwiseAlign.py command as there are rare cases this could happen. However a warning will be printed. If many warnings are printed then it is likely that step 1 did not finish correctly or you probably did not correctly specify the local PDB directory created in step 1.

- Some errors are expected; if a pair of DRPs had an error when aligning, for example if no suitable alignment could be found, these are noted as an error in their output. These will be printed to standard output but can generally be ignored, unless there are many of them (i.e. occurring more than 10% of alignments).

STEP 3: Run the cluster pipeline

```
>python clusterPipeline.py -r output/ -q drpQueryFile.txt -f similarityProduct.txt -n longerFraction.txt -d disulfideOutput.txt -s shorterFraction.txt -c 99  -t 0.7 -k 2.0 -v .01 -b 4 -l 4 -g 0.7 -p dir.des.scop.txt_1.75 -e drp_lengths.txt
```

This runs the cluster pipeline. Results are written to the directory specified by output/. Brief notes about the cutoffs:

-c: specifies the initial filtering cutoff; this should be kept at 99 to filter DRPs that are 100% identical in sequence and structure

-t: specifies the initial native overlap clustering cutoff. In the publication, this was set to 0.7. If fewer DRPs are being clustered than what was input in the publication, consider increasing this cutoff, as there will likely be fewer folds and more bias, and we have observed that these characteristics lead to misclassification of DRPs. Cutoffs can be finalized by iterative running of steps 3 and 4 and visually examining the results.

-g: We recommend this be the same value as that specified by -t

-e: set this to the drp_lengths.txt file created in step 1.

All other values are described in the usage for the script.

STEP 4: Align each cluster individually:

```
> python clusterVisAnnotation.py -r output/ -i 3 4 -c
output/processShorterSingletons_cluster_members.txt -l
output/processLongerSingletons_singleton_pairs.txt -f
output/processShorterSingletons_singleton_pairs.txt -m .7 -p localPdbDir/
```

Given cluster composition, this does a multiple structure alignment and writes out the aligned PDB files that can be viewed in a structure visualization program. In this example, its output is written to the same directory as the previous step.

Notes about the command line arguments:
-i: Specifies the exact cluster indices on which to perform this alignment.

-c, -l, -f: These are output files generated by the previous step that are read back in

-m: This should be identical to the value specified by -g in Step 3.

-p: This is the same directory that was defined in step 1.

Output:
After each step of clustering (native overlap, knottin reclustering, longer singletons, and shorter singletons), the set of clusters at that step is written to the appropriate output file (<step>_cluster_members.txt). The final cluster member file is processShorterSingletons_cluster_members.txt.

All of these files are tab-separated columns with each DRP in your input along with the index of the cluster in which it falls.

Step 4 takes these DRPs and does a final alignment, using all DRPs in each cluster. The final PDB coordinates are written out and can be viewed in a structure visualization viewer such as PyMol. This is only done over the full length of the cluster; an alignment step using overlapping disulfides (for example, in the knottin clusters) is not provided, although can be generated by adapting the code in alignDisulfides.py

Please direct questions about running the code to the corresponding author on the paper. We have done basic error checking but this is not enterprise-level software by any means so we can't account for differences in user environments, or for any idiosyncrasies that might be present in DRP PDB files that we haven't observed before.
